# Retraction: The Impact of Charlson Comorbidity Index on Mortality From SARS-CoV-2 Virus Infection

**DOI:** 10.7759/cureus.r53

**Published:** 2022-03-17

**Authors:** Jawad Ahmed, Camilo Andrés Avendaño Capriles, Natalia M Avendaño Capriles, Shivani M Mehta, Nattaliea Khan, Sheharyar Tariq, Ramsha Abbas, Sohaib Tousif, Khizer Shamim

**Affiliations:** 1 Medicine, Liaquat National Hospital and Medical College, Karachi, PAK; 2 Foundations of Clinical Research (FCR) Program, Harvard Medical School, Boston, USA; 3 Medicine, Universidad del Norte, Barranquilla, COL; 4 Medicine, Xavier University School of Medicine, Oranjestad, ABW; 5 Medicine, Ziauddin University, Karachi, PAK; 6 Medicine, Lahore Medical and Dental College, Lahore, PAK; 7 Medicine, Shifa College of Medicine, Islamabad, PAK; 8 Medicine, Ziauddin Medical University, Karachi, PAK; 9 Medicine, Ziauddin University Hospital, Karachi, PAK

This article has been retracted due to the unknown origin of the data, lack of verified IRB approval, and purchased authorships. While not listed as an author, it was discovered that Rahil Barkat wrote and coordinated the submission of this article. Mr. Barkat was involved in data theft and misuse in two recently published Cureus articles, which have since been retracted.

As the origin of this article’s data and verified IRB approval cannot be confirmed, we have made the decision to retract this article. Cureus has confirmed that the co-authors were asked by Mr. Barkat to proofread the article and provide payment in exchange for authorship. (Proofreading is an insufficient contribution to warrant authorship as defined by ICMJE.) These payments were made in the guise of “editing fees” but greatly exceed any editing fees paid to Cureus. While these authors may have been defrauded by Mr. Barkat, they remain complicit due to their lack of honest contributions to the article.

